# Heart rate adaptive maximal resolution cardiovascular magnetic resonance myocardial stress perfusion imaging at 3.0T

**DOI:** 10.1186/1532-429X-16-S1-P188

**Published:** 2014-01-16

**Authors:** David P Ripley, David M Higgins, Adam K McDiarmid, Gavin Bainbridge, Akhlaque Uddin, Ananth Kidambi, John P Greenwood, Sven Plein

**Affiliations:** 1 Multidisciplinary Cardiovascular Research Centre (MCRC) & Leeds Institute of Genetics, Health and Therapeutics, University of Leeds, Leeds,, UK; 2 Philips Healthcare, Best, Netherlands

## Background

**Figure 1:**
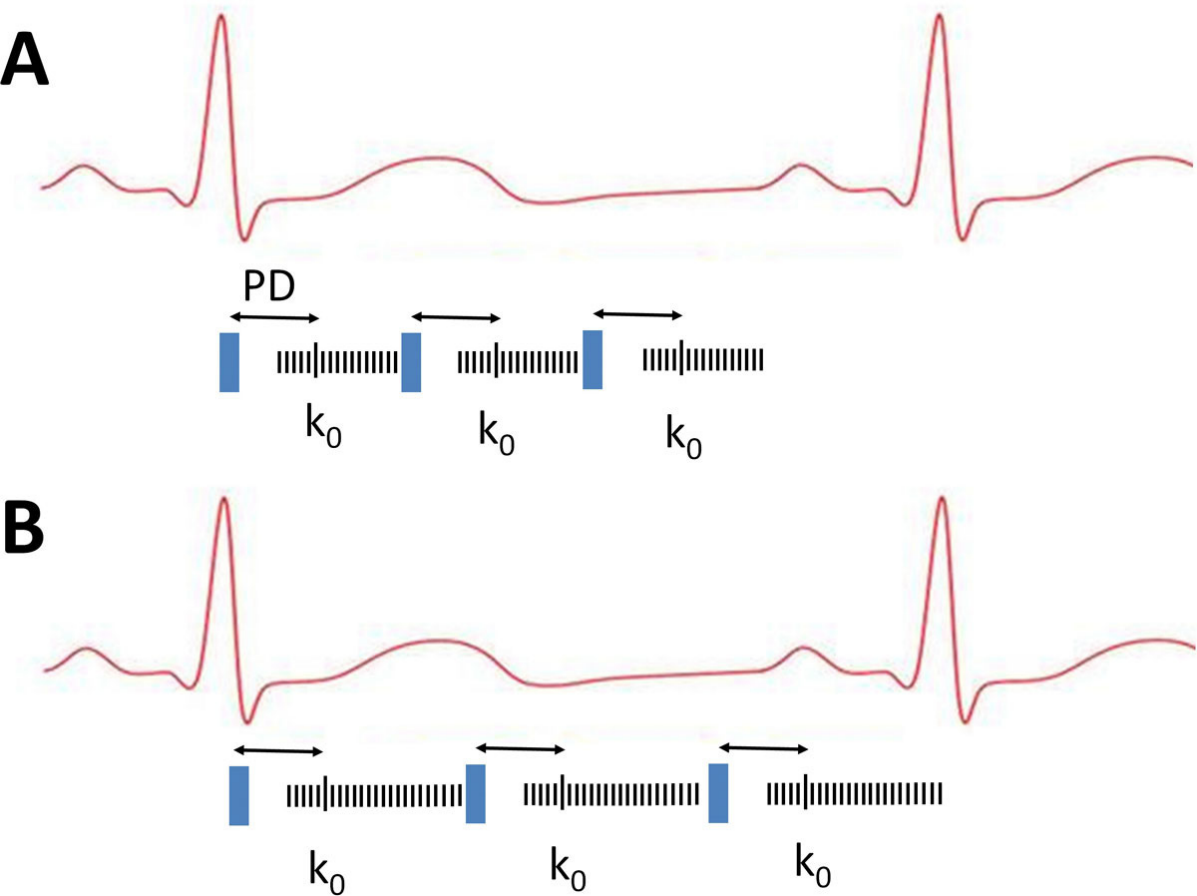
**A: Fixed resolution pulse sequence and B: Adaptive resolution pulse sequence with acquisition duration maximised for heart rate**. Blue: Pre-pulse; PD - Preparation pulse Delay time; k0: true centre of K space.

Myocardial perfusion cardiovascular magnetic resonance (CMR) with vasodilator stress has high diagnostic accuracy for detecting coronary artery disease (CAD). Current CMR perfusion pulse sequences use largely fixed acquisition parameters designed to acquire at least three slices every heart beat, optimized for the heart rates that typically occur during pharmacological stress. In patients with lower heart rates there can be a significant amount of unused potential imaging time [Figure 1]. In those with higher heart rates, acquisition with fixed parameters may not be possible at every heart beat. A more flexible acquisition scheme could optimize acquisition parameters specifically for each patient and heart rate with potential improvements in image quality or temporal resolution. We aimed to assess the feasibility of a perfusion pulse sequence which adapts to the heart rate, maximizing imaging time and acquired spatial resolution.

## Methods

A new perfusion method which automatically adapts the acquisition duration to maximise spatial resolution whist maintaining 3 slice imaging at every heart beat was developed [Figure 1]. Ten healthy volunteers and two patients underwent adenosine stress and rest perfusion CMR on two separate occasions using a 3.0T whole body scanner and dedicated 32 channel cardiac coil. On one occasion, a conventional fixed resolution perfusion sequence was used (3 short axis slices, SENSE acceleration and in-plane resolution of 2.42 × 2.42 mm). On a second occasion, the adaptive method was used. Images were evaluated blinded to the sequence and image quality graded (1 = high, 2 = adequate, 3 = poor, 4 = unusable) and DRA was measured with electronic callipers at standardized windows settings.

## Results

**Figure 2:**
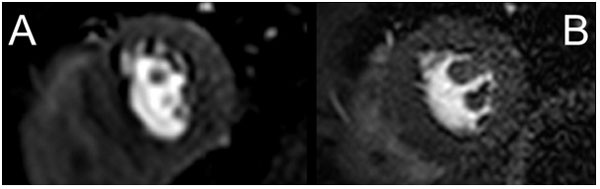
**Example perfusion first pass perfusion mid-slice images A: standard optimised fixed resolution protocol with acquired resolution of 2.42 × 2.42 mm 2 and B: adaptive resolution protocol maximising imaging time with acquired resolution of 1.74 × 1.74 mm 2 revealing less dark rim artefact**.

Adaptive perfusion CMR was feasible in all subjects. Mean stress heart rate (HR) was 89 ± 11 in the fixed resolution group and 90 ± 18 in the adaptive resolution group. There was no statistical difference in the haemodynamic data between the two groups. The standard perfusion sequence acquired in-plane resolution was 2.42 × 2.42 mm and the mean HR adaptive sequence resolution was 1.91 × 1.91 mm ± 0.41 (range 1.53-2.89)(p = 0.001). In two cases the stress HR was too high for alternate R-R interval imaging with the fixed resolution sequence resulting in alternate heart beat imaging. This did not occur with the adaptive sequence which adjusted the resolution was adapted (to 2.84 × 2.84 and 2.89 × 2.89 mm respectively). The mean DRA width was 3.0 ± 0.6 mm (95% CI: 2.57-3.51) with the standard perfusion sequence and 2.1 ± 0.6 mm (95% CI: 1.65-2.57) with the adaptive sequence (p < 0.001)[Figure 2]. There was no statistical difference in median image quality score.

## Conclusions

Optimising the use of available imaging time during CMR myocardial perfusion imaging with heart rate adaptive shot acquisition duration is feasible and improves the acquired resolution and reduces dark rim artifact whilst maintaining image quality. The effect on diagnostic performance of perfusion CMR should be investigated.

## Funding

SP is funded by a British Heart Foundation fellowship (Fs/10/62/28409) SP and JPG received an educational research grant from Philips Healthcare.

